# Reconstruction of a scoliotic spine using a three-dimensional medical ultrasound system

**DOI:** 10.1186/1748-7161-10-S1-P17

**Published:** 2015-01-19

**Authors:** Quang N Vo, Edmond H M Lou, Lawrence H Le

**Affiliations:** 1 University of Alberta, Canada; 2 Ho Chi Minh City University of Technology, Vietnam

## Objective

Ultrasound imaging has been used to measure the coronal curvature and vertebral rotation on children with scoliosis. However, those studies still use the two-dimensional information to report the three-dimensional (3D) deformity. To truly describe the 3D curvature of spine, a 3D spinal image is needed. This study reported a preliminary experiment to reconstruct a 3D spinal curvature from both in-vitro and in-vivo data acquired from a medical ultrasound system.

## Materials and methods

A medical ultrasound system and a 4.0 MHz convex probe with a built-in positioning system were used. The positioning system is essential to reconstruct the 3D image. Three different types of spine were scanned: i) two Sawbones spine phantoms: one was a normal spine without any vertebral rotation and the other was set as a moderate curve with certain vertebral rotation, ii) a non-scolitotic spine from a 33-year-old male healthy volunteer and iii) a scoliotic spine from a 16-year-old female AIS patient with right thoracic (14°) and left lumbar (21°). The phantoms were scanned from T6 to L5, and the subjects were from T1 to L5 in standing positions.

After acquisition, the 3D ultrasound data was fed to an in-house developed program. To accelerate the reconstruction process, the program first eliminated the overlapping data. The manual determination of region of interest followed to further eliminate unnecessary information. The median filter was then applied to reduce the noise. The voxel-based approach was used to perform the volume formation. Smoothing was the last step before volume rendering. This proposed method was first applied to the reconstruction of 3D spine.

## Results

The 3D image of the normal spine phantom provided the most detailed and the clearest landmarks including laminae, spinous processes and transverse processes. Meanwhile, the scoliotic phantom showed lower image quality with some noise. Some missing data appeared on the spinous process from T10 to L5. The spines reconstructed from the in-vivo data had more noise, lower image quality and blurs occurred at the lumbar region. Fortunately, all the important landmarks such as laminae and transverse processes could be visualized in the 3D images.

## Conclusions

The results demonstrated that it was feasible to use ultrasound to reconstruct the posterior section of spine three-dimensionally. The recognized landmarks are important for future studies as they are the major features which can be used to estimate the severity of scoliosis in ultrasound images. Further image processing is needed to improve image quality.

